# Measuring the Sense of Agency: A French Adaptation and Validation of the Sense of Agency Scale (F-SoAS)

**DOI:** 10.3389/fpsyg.2020.584145

**Published:** 2020-10-08

**Authors:** Jean-Christophe Hurault, Guillaume Broc, Lola Crône, Adrien Tedesco, Lionel Brunel

**Affiliations:** ^1^Laboratory Epsylon EA 4556, Department of Psychology, University Montpellier 3, Montpellier, France; ^2^Centre de Recherches sur la Cognition et l’Apprentissage UMR7295, University of Poitiers, Poitiers, France

**Keywords:** sense of agency, judgment of agency, questionnaire, French adaptation, gender differences, invariance property

## Abstract

Sense of Agency (SoA) is the subject of growing attention. It corresponds to the capacity to claim authorship over an action, associate specific consequences with a specific action, and it has been claimed to be a key point in the development of consciousness. It can be measured using the Sense of Agency Scale (SoAS), originally proposed by Tapal et al. (2017), who distinguished it into two-factor: Sense of Positive Agency (SoPA) and Sense of Negative Agency (SoNA). This study reports on the first adaptation of the SoAS into another language, French. For this French version of the Sense of Agency Scale (F-SoAS), we analyzed responses from a sample of 517 native French-speakers. Our results indicate that the scale has good psychometric properties. Factor analysis confirms the same two-factor model as Tapal et al. (2017). However, some items were removed due to insufficient loadings with factors, leading to a short version of the scale (7-item). Furthermore, we observed gender differences that are consistent with findings in the literature. Specifically, women report higher SoNA scores and lower SoPA scores than men. We conclude by discussing possible uses and future directions for the scale.

## Introduction

### Definition and Theory

Sense of Agency (SoA) refers to the subjective perception of being an agent, i.e., “I” am the one who is causing some event in the world to occur (Haggard and Chambon, 2012). The elements leading to this feeling are becoming better-known, for example, changes in our environment are likely to elicit a SoA if they occur close in time and space to our action (Moore et al., 2012; Moore and Obhi, 2012). In recent years, the issue has been the subject of substantial research within cognitive science (e.g., Pacherie, 2007; Aarts et al., 2012; Friston, 2012). The phenomenon is a fundamental aspect of consciousness, as it allows us to distinguish between those sensory consequences that we cause, and those that are externally generated.

Initially, SoA was hypothesized to be solely based on motor processes, notably the comparator process (Blakemore et al., 2000; Tsakiris et al., 2005). However, empirical evidence shows that non-motor processes, such as causal attribution, can also generate SoA (Wegner, 2004). More recently, it has been theorized to be based on the integration of both motor and non-motor processes, known as multiple (or optimal) cue integration theory (Synofzik et al., 2008; Moore and Fletcher, 2012; Vosgerau and Synofzik, 2012; Pacherie, 2013). This new approach can reconcile many heterogeneous and seemingly unrelated findings from recent SoA studies (Synofzik, 2015). SoA appears to be a multisensory process; different cues are weighted, enabling a flexible, reliable experience of agency in different contexts (Gallagher, 2012; for a contradictory review, see Reddy, 2019).

According to cue integration theory, SoA can be divided into a feeling of agency and a judgment of agency. Feelings of agency refer to the (motor-processing) “low-level” feeling of being the agent of an action while judgments of agency refer to the (cognitive-processing) “high-level” judgment of being an agent. These two aspects are assessed differently: “indirect” measures are used for feelings while “direct” measures are used for judgments (Haggard, 2017).

### Measures of SoA

With respect to indirect measures, intentional binding has become one of the most, if not the most, promising phenomena used to study SoA (Haggard and Tsakiris, 2009). The concept refers to the perceived compression of the time interval between an intentional action and its outcome, compared to an involuntary action (Moore and Obhi, 2012). Binding can be measured by various methods: timing an individual action or outcome event with a Libet clock (Haggard et al., 2002), delay judgment (Kawabe et al., 2013), or the direct estimation of the interval between an action and its outcome (Engbert et al., 2007). The magnitude of the effect is considered to be an indicator of the level of SoA (e.g., Caspar et al., 2016; Khalighinejad et al., 2016). Various other phenomena have been associated with SoA: sensory attenuation (Hughes et al., 2013), spatial binding (Kirsch et al., 2016), and motivation from control (Eitam et al., 2013). However, indirect measures have been shown to be sensitive to experimental manipulations of people’s beliefs about their agency (Desantis et al., 2011; Berberian et al., 2012).

Direct measures principally consist of rating scales or self-report questionnaires about agency in a specific experimental event. They require participants to reflect upon his/her agency and answer questions such as “Did I do that?” or “To what degree did I feel that my action caused that specific event?” (e.g., Wegner and Wheatley, 1999; Aarts et al., 2005). Although such measures concern “local,” highly contextualized judgments of agency, researchers have often found inconsistent, or no, correlations with indirect measures of SoA (Dewey and Knoblich, 2014). The latter observation makes it more difficult to accurately identify markers of agency and to generate a conceptual consensus regarding cognitive mechanisms.

### A New Measure for the JoA

In this context, Tapal et al. (2017) proposed that the absence of clear correlations between direct and indirect measures of SoA could be due to “the lack of a valid and reliable tool for measuring […] decontextualized, cross situational (or “chronically held”) cognitions.” The latter refers to the broad definition of the judgment of agency in multiple cue integration theory. Although numerous scales have been developed to measure the judgment of agency, they have been limited to local events and do not rate global agency. Thus, Tapal et al. (2017) sought to directly assess general SoA (i.e., across different situations) by developing a psychometric scale, the Sense of Agency Scale (SoAS). This scale was derived from a factor analysis of a large number of items based on a broad review of the various ways in which agency has been described in the psychological literature (for more details on the developmental process, see Tapal et al., 2017). The SoAS seems to be an appropriate way to assess general SoA. Moreover, to the best of our knowledge, the only other scale is the Sense of Agency Rating Scale (SOARS; Polito et al., 2013), which assesses the specific case of global SoA during hypnosis. However, the SoAS was only validated in Hebrew with an Israel population. Thus, there is a need to adapt it in other languages in order to provide clear evidence of the good psychometric properties of the scale.

The SoAS comprises two-factor, representing opposite aspects of the SoA: the Sense of Positive Agency (SoPA) and the Sense of Negative Agency (SoNA). The SoPA represents the level of control over the body, mind, and environment felt by an individual. Conversely, the SoNA represents the lack of control over the body, mind, and environment felt by an individual. In their study, Tapal et al. (2017) assessed the validity of the scale by comparing it to other, related concepts, such as self-efficacy (Bandura, 1977), locus of control (Rotter, 1966), sense of control (Lachman and Weaver, 1998), and endorsement of determinism of fatalism (Paulhus and Carey, 2011). They confirmed that the SoAS measures a concept that is distinct from a general or specific, belief in self-efficacy, and from a belief of having control over obtaining a desired outcome (locus of control). In addition, their compared SoAS with concepts, such as free will and determinism belief, provide additional support for the distinction between the two-factor making up the SoAS. Their findings suggest that SoPA is about personal autonomy and responsibility over our actions while SoNA is about fatalism and existential helplessness (for more details on these distinctions, see Study 2 of Tapal et al., 2017).

This two-factor model is compatible with recent neuroscientific evidence that supports the existence of an anatomical differentiation between agency judgments (Nahab et al., 2011; Sperduti et al., 2011). Additionally, results of Tapal et al. (2017) comparison of SoNA and philosophical fatalism measures are coherent with the literature on SoA and learned helplessness (for a short review, see Peterson, 2010), new neuroscientific evidence about learned helplessness (Maier and Seligman, 2016), and experimental manipulations (Karsh et al., 2018). Taken together, these studies advocate for an interpretation of SoNA as a special, and generally negative case of not having control over the environment, which is cross-situational (or “chronic”) rather than focused on a specific situation (or aspect of the experience of agency).

### Gender Effects on SoA

Other explorations of the two SoA factors indicate that it may differ across genders (Valås, 2001; Jones et al., 2008; Jejeebhoy et al., 2010; Donald et al., 2017). Overall, results show that women report lower levels of SoA than men. However, group comparisons must be interpreted with caution and it is important to consider the implications of measurement invariance. This measure refers to the extent to which the content of each item of a scale is perceived and interpreted in the same way across samples (Byrne and Watkins, 2003). If this is not controlled, group comparisons are likely to be misleading and potentially artifactual (e.g., Byrne and Stewart, 2006). Only when it is controlled can meaningful comparisons of statistics (such as means) be made.

In sum, global SoA, as measure by the SoAS, can be divided into two aspects: one concerning the judgment of having control (SoPA) and the other concerning the judgment of being existentially helpless (SoNA). Both aspects reflect perceptions and cognitions regarding SoA in general. As Tapal et al. (2017) argue, SoAS may “enable some structuring of the complicated and often-conflicting pattern of findings obtained by direct and indirect measures of SoA and of the relationships among them by enabling consistent measurement of individual differences in (or situational effects on) ‘global’ sensing of agency.”

### Present Research

As advocated above, it is clearly important to continue to validate these measures in order to investigate links between implicit and explicit SoA. The SoAS is the first scale to propose a measure for global SoA. However, this scale is only accessible in Hebrew and needs adaptation into other languages to confirm its two-factor model. Therefore, the main objective of our work was to first adapt and validate a French version of the SoAS (F-SoAS). By factor analysis (EFA and CFA), our study aimed to validate the two-factor structure of the F-SoAS, in a large sample of French-speaking adults (*N* = 517), and examined its reliability and construct validity. Another aim of our study was to investigate the effect of gender on F-SoAS scores. First, by assessing whether this measurement of agency functions similarly across genders (i.e., same perception of items/equivalent response process) and second, by comparing SoAS scores for women and men.

## Materials and Method

### Translation and Adaptation

In order to adapt the original material into French, we followed current standards for translating and adapting tests (American Psychological Association, American Educational Research Association and National Council on Measurements Used in Education, 1954; American Educational Research Association, American Psychological Association, and National Council of Measurement in Education-AERA, APA, and NCME, 2014; International Test Commission, 2014). First, approval was obtained from the authors to adapt their original material. They also provided instructions to help us better-understand how the SoAS was constructed and help us to replicate it. Then, two independent translations into French were prepared by native French speakers with expertise in psychology. Here, the aim was to fit the semantic, conceptual, and cultural properties of the source material to the target test. Both translations were then back-translated by two other independent bilingual (English/French) translators. Then, original and back-translated versions of the SoAS were compared. This resulted in a preliminary, unified version that was approved following discussion by a representative panel of experts in the field. The final, French version did not differ from the original with respect to item titles, response scale, or instructions.

### Procedure

The scale was initially distributed online *via* our university digital workspace and Facebook groups, using our laboratory’s web service, Epsylab.^1^ However, in order to expand the sample, we decided to distribute a paper version of the scale to other first-year bachelor’s students at our university. To estimate the consistency of the measure (reliability), we used a test-retest method. Notably, we asked the same sample to complete the same questionnaire again 2 months later.

### Participants

Given the need to use a fairly large sample to accurately adapt and validate an instrument (Gudmundsson, 2009) and the recommendation to use at least 10 subjects per item (Schumacker and Lomax, 2004; Brown, 2015; Kline, 2015), our convenience sample was composed of 517 native French speakers (430 females and 87 males; mean age 23.04 ± 8.66). Most were students (90.91%) or had graduated from high school (73.89%). Participation, either online (224 subjects) or on paper (293) was voluntary and participants were informed about the confidentiality/anonymity of their data. In order to evaluate stability, participants who completed it online received an email 2 months later inviting them to complete the scale again. Sixty-two subjects (11.99% of the sample) responded to this request. There were no incentives for participation, and ethical guidelines from the Helsinki Declaration (World Medical Association, 2018) were followed. The only exclusion criteria were an incomplete answer to one of the questions – this excluded 62 participants (11.99%).

### Measures

The French version of the 13-item SoAS (F-SoAS) was used in our study. According to the factor structure of the original construct, SoA is divided into SoPA (i.e., the feeling of having control) and SoNA (i.e., the feeling of being existentially helpless). The response to each item was recorded on a 7-item Likert scale ranging from 1 (strongly disagree) to 7 (strongly agree). The English translation of the SoAS can be found in Table 1. In addition, participants provided socio-demographic information: gender, age, and academic achievement.

**Table 1:** Factor loadings of items after rotation.

SoAS items	SoPA	SoNA
01. I am in full control of what I do	**0.58**	0.10
02. I am just an instrument in the hands of somebody or something else	0.20	**0.27**
03. My actions just happen without my intention	0.17	**0.55**
04. I am the authors of my actions	**0.40**	0.20
05. The consequences of my actions feel like they do not logically follow my actions	0.15	**0.36**
06. My movements are automatic – my body simply makes them	−0.32	**0.48**
07. The outcomes of my actions generally surprise me	0.09	**0.48**
08. Things I do are subjects only to my free will	**0.67**	−0.07
09. The decision whether and when to act is within my hands	**0.61**	0.03
10. Nothing I do is actually voluntary	0.23	**0.31**
11. While I am in action, I feel like I am a remote controlled robot	−0.06	**0.49**
12. My behavior is planned by me from the very beginning to the very end	**0.26**	0.04
13. I am completely responsible for everything that results from my actions	**0.45**	0.06
**SS loading/Eigenvalue**	**1.91**	**1.45**
**Cumulative Variance**	**0.15**	**0.11**

English translation of items from Tapal et al. (2017); SoPA, Sense of Positive Agency; SoNA, Sense of Negative Agency. The higher of the two loadings appears in bold.

### Statistical Analysis

The sample was split randomly into two independent samples. The first random sample (*N* = 259) was used to perform an Explanatory Factor Analysis (EFA). The second (*N* = 258) to test the measurement model through Confirmatory Factor Analysis (CFA). Finally, we used the complete sample (*N* = 517) to analyze constructs validity, construct stability, constructs equivalence, and gender differences on SoAS scores.

The Explanatory Factor Analysis and Confirmatory Factor Analysis were run using the R package “psych” (Revelle, 2015) and “lavaan” (Rosseel, 2012), followed by checks of construct validity, stability, equivalence, and gender differences conducted using JASP version 0.11.1.

#### Exploratory Factor Analysis

In order to determine whether the EFA was suitable to perform on the first random sample (*N* = 259), we applied the Kaiser-Meyer-Olkin (KMO) test of sampling adequacy and the Bartlett’s test of sphericity. The KMO indicates the ratio of the squared correlation between variables to the squared partial correlation between variables (Field, 2009). The KMO statistics vary from 0 to 1. A value of 0 indicates that the sum of partial correlations is large relative to the sum of correlations, indicating diffusion in the pattern of correlations; therefore, the factor analysis is likely to be inappropriate. A value close to 1 indicates that patterns of correlations are relatively compact and factor analysis should yield distinct and reliable factors. The lowest acceptable limit being 0.50 (Field, 2009). The Bartlett’s test of sphericity examines the null hypothesis that variables in the population correlation matrix are uncorrelated. A significant value of *p* indicates that variables (i.e., items) are sufficiently related and therefore suitable for structure detection.

To evaluate the probability of this sample to have been drawn from a multinormal population, we used the Mardia’s test (Mardia, 1970, 1974). If this test shows a significant value of *p*, indicating a deviation from normality, it is recommended using the Principal Axis Factor rather Maximum Likelihood as appropriate estimator. The parallel analysis was performed to indicate the best suitable factor solution based on the eigenvalue of the actual data higher than their corresponding random eigenvalue (Horn, 1965).

Then, the EFA was performed in this sample, with oblique rotation (OBLIMIN), to verify the underlying factor structure of the 13-item F-SoAS. Oblique rotation was used when it is assumed that the underlying factors are correlated to each other. A loading of 0.40 in one factor and a difference of 0.30 with loadings on the other factors were used as the cutoff for inclusion (O’connor, 2000).

### Confirmatory Factor Analysis

Because CFA will be conducted on a different subset, and as the model proposed by the EFA could change the variables (due to removed items), another Mardia’s test has to be done to assess whether the second random sample (*N* = 258) was likely to be drawn from a multinormal population. As recommended, a significant value of *p*, indicating a deviation from normality, would lead us to use the Maximum Likelihood Robust (MLR) as appropriate estimator.

The Confirmatory Factor Analysis was conducted on this subset to confirm the model of the F-SoAS identified through the EFA. According to Hu and Bentler (1999), a model shows adequate fit when: (1) Root Mean Square Error of Approximation (RMSEA) ≤ 0.06; (2) Standardized Root Mean Square Residual (SRMR) ≤ 0.08; and (3) Comparative Fit Index (CFI) and Tucker–Lewis Index (TLI) ≥ 0.95. It is worth noting that such criteria are only a guideline, and that values close to standards can be accepted (Bollen, 1989); for example, a CFI-TLI > 0.90 or an RMSEA up to 0.10 (Kenny et al., 2015).

#### Construct Validity

Construct validity was evaluated, on the complete sample (*N* = 517), by examining the standard factor loading (SFL) for each item, along with Composite Reliability (CR) and Average Variance Extracted (AVE; Raubenheimer, 2004). Concerning the SFL, the cutoff for the factor loading of each item with its scale was set at 0.50 (Hair et al., 2006). Concerning the CR index, we computed Cronbach’s alpha (Cronbach, 1951) but, above all, Raykov’s *ω* (Raykov, 2001), Bentler’s *ω*^2^ (Bentler, 2009), and McDonald’s *ω*^3^ (McDonald, 1999) coefficients, which proved to be much more efficient (Trizano-Hermosilla and Alvarado, 2016). Most authors recommended that CR should be above 0.70 even if 0.60 is still indicates sufficient (Carlson and Herdman, 2012). Finally, AVE should be above 0.50 (Fornell and Larcker, 1981).

#### Construct Stability

Construct stability was calculated, on the complete sample (*N* = 517), using a test-retest analysis, based on the 62 subjects who filled in the questionnaire for a second time, 2 months later. For this analysis, we used the following indexes: (1) The Intraclass Correlation Coefficient (ICC3k). This evaluates two-way mixed effects based on means of multiple measurements and on absolute agreement. Stability is good when over 0.75 (Koo and Li, 2016). (2) Paired *t*-tests and Pearson’s correlations for each factor between the first response (T0) and the second response (T1).

#### Construct Equivalence Across Genders

Construct equivalence was estimate, on the complete sample (*N* = 517), using measurement invariance. It allows datasets collected from different groups, or sociocultural contexts, to be meaningfully compared.

First, we assessed the internal structure of the measurement model among women and men independently. In order to do so, two separated CFA with MLR estimator were performed on a female sample (*N* = 430) and a male sample (*N* = 87).

Second, we test for measurement invariance by a series of hierarchical steps based on multigroup CFA models. It begins with the establishment of a baseline model in each group followed by tests for equivalence across groups at increasingly restricted levels. The first step (configural invariance) aims to test whether each group has the same number of dimensions, and patterns of fixed and free parameters (Bollen, 1989). The next step seeks to assess whether factor loadings for latent variables (i.e., weak invariance) are also invariant across groups. If this condition holds, the next step is to test whether intercepts (i.e., strong invariance; Widaman and Reise, 1997), then residuals (i.e., strict invariance) and, finally, means, variances, and covariances of latent variables (i.e., structural invariance) are invariant across groups.

The evaluation was made with the most-commonly-used chi-square test (Cochran, 1952). A non-significant difference (*p* > 0.05) between a less and more constrained model indicates that the new constraint does not degrade the quality of adjustment. In other words, the assumption of between group invariance remains plausible at the finest level of observation likely to vary. In addition, we used the Bayesian Information Criterion (BIC) and the Akaike’s Information Criterion (AIC) to compare the relative fit of models (Wicherts and Dolan, 2004). A lower BIC/AIC indicates a better trade-off between model fit and model complexity.

#### Gender Differences on F-SoAS Scores

Gender differences between F-SoAS scores were examine, on the complete sample (*N* = 517), using an ANOVA. First, for F-SoAS as a global score and then for scores for each factor with gender as a between-subjects factor.

## Results

### Exploratory Factor Analysis

On the first random sample (*N* = 259), the KMO value for this data was 0.78, which is correct (values over 0.5 are considered proper) and KMO values for each individual item were larger than the cutoff of 0.50. Concerning the Bartlett’s test of sphericity, the approximate Chi-Square obtained was significant, *χ*^2^(78, *N* = 259) = 493.16 and *p* < 0.001, meaning, in accordance with KMO, that the correlation matrix is suitable for factor detection. As Mardia test was statistically significant for skewness and kurtosis (Mardia skewness = 25.72, *χ*^2^(455, *N* = 259) = 1110.19, *p* < 0.001; Mardia kurtosis = 224.63, *Z* = 12.07, *p* < 0.001), we used the Principal Axis Factor method to perform the EFA.

**Figure 1:**
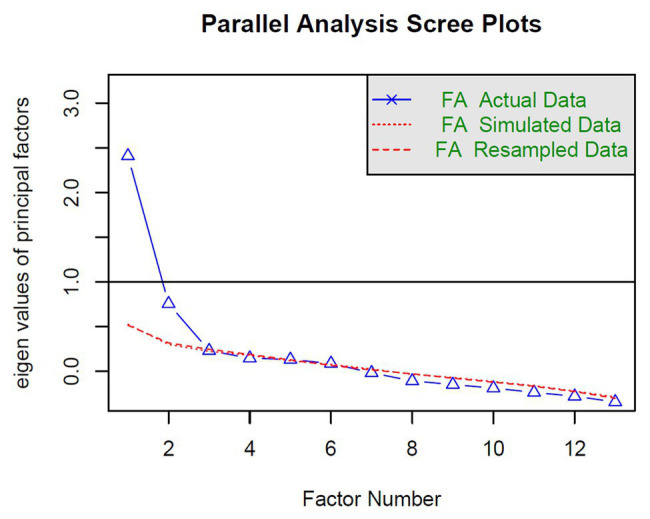
Results of parallel analysis showing the factor number on the horizontal axis and eigenvalue of the vertical axis. Eigenvalues for actual data were: 2.41 (0.52), 0.75 (0.31), 0.23 (0.24), 0.14 (0.18), 0.13 (0.12), 0.08 (0.08), −0.02 (0.02), −0.11 (−0.03), −0.15 (−0.08), −0.19 (−0.11), −0.24 (−0.17), −0.28 (−0.25), −0.35 (−0.31), with their corresponding random eigenvalue in brackets.

The parallel analysis of the EFA suggested a two-factor solution based on the eigenvalue of the actual data that are greater than their corresponding random eigenvalue (see Figure 1). The EFA was performed on a two-factor solution (see Table 1). For the factor 1 (SoPA), in accordance with the item inclusion criteria (see Materials and Methods section), we removed: item 4 (cross-loading < 0.30) and item 12 (loading < 0.40). For factor 2 (SoNA), we removed: item 2 (loading < 0.40), item 5 (loading < 0.40), item 6 (cross-loading < 0.30), and item 10 (loading < 0.40). Thus, leading to a 7-item scale, with four items in the SoPA factor and three items in the SoNA.

### Confirmatory Factor Analysis

On the second random sample (*N* = 258), the Mardia test was statistically significant for skewness and kurtosis (Mardia skewness = 26.16, *χ*^2^(455, *N* = 258) = 1124.97, *p* < 0.001; Mardia kurtosis = 226.34, *Z* = 12.75, *p* < 0.001). Consequently, the CFA was based on the Robust Maximum Likelihood Robust (MLR) estimator (Satorra and Bentler, 1994).

**Figure 2:**
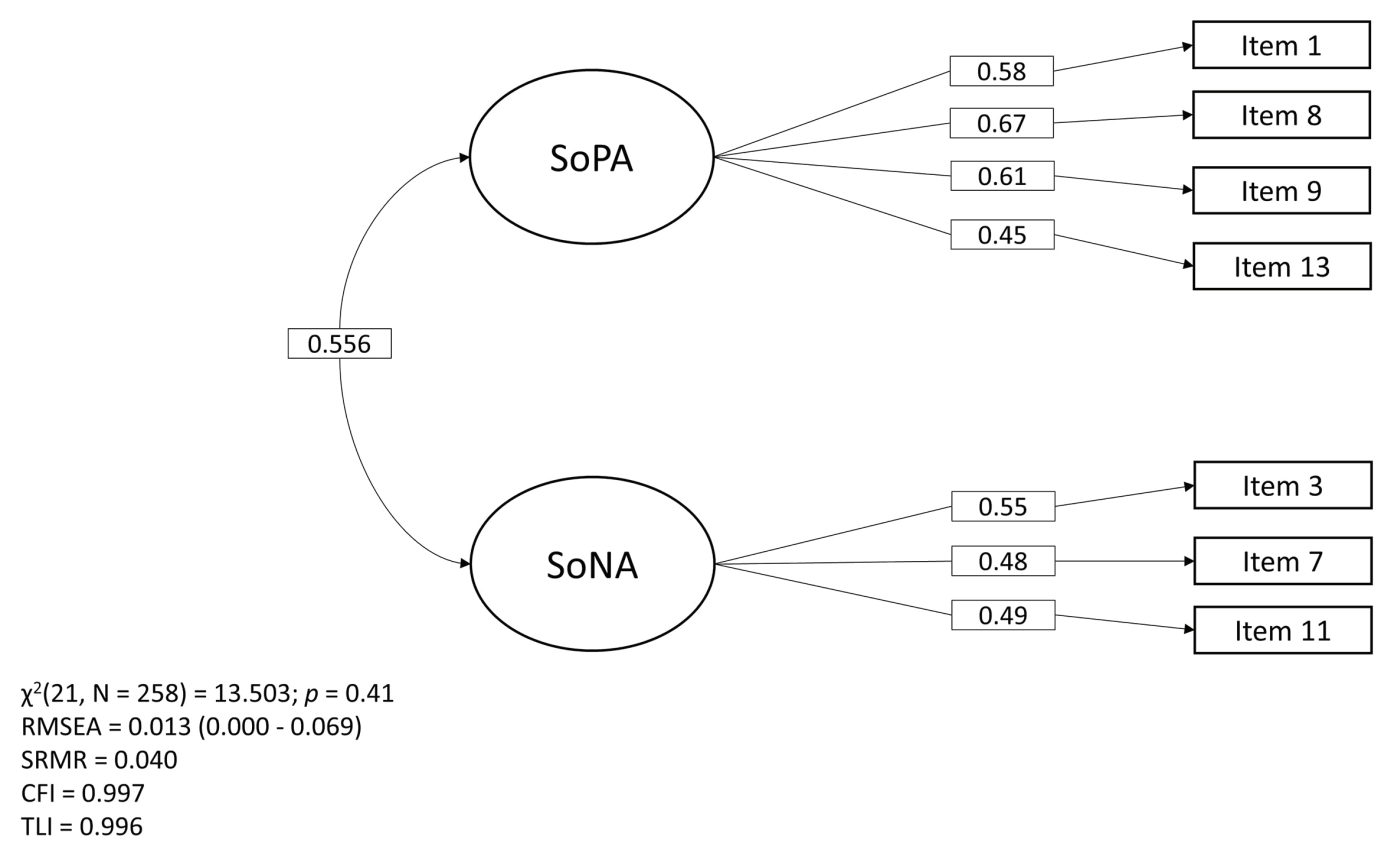
The structure and parameters of the two-factor confirmatory model. SoPA, Sense of Positive Agency; SoNA, Sense of Negative Agency. The figure presents (from left to right) the correlation between the two latent variables and standardized residuals. Variances of both latent variables were constrained to 1.

The model, identified through the EFA, fitted the data adequately: *χ*^2^(21, *N* = 258) = 13.503, *p* = 0.41; CFI = 0.997; TLI = 0.996; RMSEA = 0.013 (90% CI = 0.000, 0.069) and SRMR = 0.040. Covariance between factors was *β* = 0.556 (*p* < 0.001). Thus, every index is in accordance with the criteria. Therefore, we conclude that the model is an adequate fit to our data (see the Statistic Analysis section for details on criteria). Figure 2 shows the model diagram with parameter estimates.

### Construct Validity

On the complete sample (*N* = 517), the Mardia test was statistically significant for skewness and kurtosis (Mardia skewness = 26.16, *χ*^2^(455, *N* = 517) = 1124.97, *p* < 0.001; Mardia kurtosis = 226.34, *Z* = 12.75, *p* < 0.001).

The standard factor loading was above 0.50 for all items, except: item 7 (SFL = 0.49), item 11 (SFL = 0.44), and item 13 (SFL = 0.44), which were borderline. Concerning the CR values, Cronbach’s alpha values, computed for each factor, were 0.64 for the SoPA and 0.55 for the SoNA. The overall alpha value of the SoAS was 0.66. It was above the cutoff (0.60) for the overall alpha (SoAS) and for the SoPA factor but not for the SoNA factor, indicating medium reliability (Stormer et al., 1999). Besides, McDonald’s omega (McDonald, 1999) was ω^3^ = 0.65 for SoPA and ω^3^ = 0.53 for SoNA. It was above the cutoff (0.60) for the SoPA factor but not for the SoNA factor. None of the alpha and omega indices were above the threshold of 0.70, indicating insufficient reliability. Similarly, the AVE for each factor did not exceed the cutoff (0.50), also indicating unsatisfactory construct validity (SoPA: AVE = 0.32; SoNA: AVE = 0.32).

### Construct Stability

Concerning test-retest analysis, total scores did not change significantly over the 2-month period [*t*(61) = −0.40; *p* = 0.69], with a mean of 35.55 (±5.62) for the first measurement and 35.77 (±5.53) for the second measurement. The ICC was 0.82 (*p* < 0.001), indicating good test-retest stability. SoPA scores also remained stable over the period [*t*(61) = −1.19; *p* = 0.24] with means of 18.53 (±4.12) for the first measurement and 19.05 (±3.92) for the second measurement. The ICC was 0.78 (*p* < 0.001), indicating good test-retest stability. SoNA scores also remained stable over the period [*t*(61) = −0.86; *p* = 0.39] with means of 6.98 (±2.79) for the first measurement and 7.27 (±2.88) for the second measurement. Here, the ICC was 0.72 (*p* < 0.001), indicating good test-retest stability. Additionally, Pearson’s correlations were: *r* = 0.69 (*p* < 0.001) for the total F-SoAS score; *r* = 0.64 (*p* < 0.001) for the SoPA; and *r* = 0.56 (*p* < 0.001) for the SoNA. Overall, our reliability analysis support the interpretation that F-SoAS does indeed estimate people’s cross-situational SoA and not local, situation-dependent, SoA.

### Construct Equivalence

Concerning the female sample, the Mardia’s test was statistically significant for skewness and kurtosis (Mardia skewness = 19.86, *χ*^2^(455, *N* = 430) = 1422.99, *p* < 0.001; Mardia kurtosis = 227.90, *Z* = 17.27, *p* < 0.001). Thus, CFA was based on the Robust Maximum Likelihood estimator. The model identified through the EFA fitted the female sample adequately: *χ*^2^(13, *N* = 430) = 20.470, *p* = 0.084; CFI = 0.975; TLI = 0.954; RMSEA = 0.037 (90% CI = 0.000, 0.064) and SRMR = 0.038. The covariance between factors was *β* = 0.443 (*p* < 0.001). Every index was in accordance with the criteria. Therefore, we concluded that the model is an adequate fit to the female sample.

Concerning the male sample, the Mardia’s test was statistically significant for skewness and kurtosis [Mardia skewness = 56.89, *χ*^2^(455, *N* = 87) = 824.98, *p* < 0.001; Mardia kurtosis = 216.24, *Z* = 5.02, *p* < 0.001]. Thus, CFA was based on the Robust Maximum Likelihood estimator. The model identified through the EFA fitted the male sample adequately: *χ*^2^(13, *N* = 87) = 20.367, *p* = 0.086; CFI = 0.933; TLI = 0.891; RMSEA = 0.082 (90% CI = 0.000, 0.147) and SRMR = 0.062. The covariance between factors was *β* = 0.747 (*p* = 0.002). Almost every index was in accordance with the criteria only TLI and RMSEA falls outside the criteria but was very close. Therefore, we concluded that the model is an adequate fit to the male sample.

Following, the measurement invariance was evaluated. Its results are presented in Table 2. The first point to note is that, except for the structural model, the more constrained the model, the more AIC and BIC decreased, and best fit was observed for the F-SoAS two-factor structure. Second, the value of *p* was non-significant for each invariance test, except for the structural model which remains, it should be recognized, a fairly demanding condition rarely reach in practice (Gana and Broc, 2019). Therefore, apart from this consideration, it may be stated that the hypothesis of equivalence of the measurement model across genders is demonstrated.

**Table 2:** Results of measurement invariance across genders.

	*χ*^2^_SB_	Df	AIC	BIC	Δ*χ*^2^_SB_	ΔDf	*p*
Configural	43.32	26	12,161	12,348	−	−	−
Weak	46.84	31	12,154	12,320	3.16	5	0.67
Strong	53.60	36	12,151	12,295	6.87	5	0.23
Strict	64.17	43	12,148	12,262	5.47	7	0.60
Structural	80.76	48	12,154	12,248	16.29	5	0.01

The MLR is used as the estimator. *χ*^2^SB, Satorra-Bentler scaled chi-square; AIC, Akaike’s information criterion; BIC, Bayesian information criterion.

### Gender Differences

We found a significant effect of gender with respect to the global score with *F*(1) = 8.77, *p* = 0.003, and *η*^2^ = 0.02. Concerning individual factors, the results show a significant effect of gender for both the SoPA, with *F*(1) = 4.07, *p* = 0.04, and *η*^2^ = 0.01 and the SoNA, with *F*(1) = 9.12, *p* = 0.003, and *η*^2^ = 0.02. Women scored lower than men, both for the global score (women = 36.16 ± 5.89; men = 38.10 ± 5.98) and for the SoPA (women = 19.44 ± 4.06; men = 20.43 ± 4.47), but higher than men on the SoNA (women = 7.28 ± 2.74; men = 6.32 ± 2.51).

## Discussion

As noted in the introduction, despite renewed interest in SoA, questions remain about how to measure it. In this context, the principal aim of the current study was to extent the use of the SoAS, by validating the first adaptation of the scale into another language, the F-SoAS, which we call the F-SoAS. In addition, the study aimed at testing gender differences among SoAS scores as found in the literacy of gender effect on SoA.

### Psychometrical Findings

Our results show that the F-SoAS has quite good psychometric properties – with very good fit, acceptable-to-good saturations, test-retest reliability, and structure equivalence across genders, but very poor construct validity –, and provide overall encouraging results. Both EFA and CFA demonstrated results in line with the two-factor measurement model validated by Tapal et al. (2017), thus strongly supporting the idea that global SoA is multidimensional. Notwithstanding, the low explained variance (28% in total, with 15% for the SoPA and 11% for the SoNA; see Table 1), as well as the poor construct validity, indicate an important measurement error. Hence, the scale does not yet capture enough true variance and needs improvement.

Following the EFA, we removed five items from the original 13-item SoAS because of insufficient loadings, resulting in a short-form of the F-SoAS where each dimension still encompasses a sufficient provision of items (three minimum). Besides, the low number of items (7-item scale) makes the scale more easily integrated into research studies, questionnaires, and less complex to analyze.

Concerning such removing of several items, some feedback after administering the test has proved helpful to understand to what extent the translation or the cultural context had an impact on the response process. For example, concerning the item 02 (*I am just an instrument in the hands of somebody or something else*), it is possible that the idea of being guided by an “invisible” hand could echo different beliefs according to people (e.g., gods, conspiracy…). Concerning the item 04 (*I am the authors of my actions*), the term “author” could be seen as ambiguous and encompassing positive or negative realities (e.g., author of a crime or author of an artistic work). Concerning the item 05 (*The consequences of my actions feel like they do not logically follow my actions*), it is possible that the use of the term “feel” and “logically” makes confuse the understanding of the sentence. Concerning the item 6 (*My movements are automatic – my body simply makes them*), the two distinct parts of the sentence could yield different meanings. The term “automatic” referring to the lack of control and the term “simply” referring to an ease of control. Concerning the item 10 (*Nothing I do is actually voluntary*), our adaptation in French could have made the sentence hard to understand because we used a double negative sentence. Finally, concerning the item 12 (*My behavior is planned by me from the very beginning to the very end*), the interpretation could be understood more or less literally depending on the subjects.

The results are consistent with the Hebrew version of the SoAS, which suggests that there might be only a small (or no) impact of culture on global SoA. However, this assumption should be explored in more detail in further studies as culture could have complex impact on the integration and the perception of external causes in their attributions judgments (for example, between Asian and Western culture, see Choi et al., 1999; Mezulis et al., 2004). Thus, subsequent studies investigating more structured cognitive interviews with compared qualitative analysis between countries should be of interest to better understand cross-cultural differences in the adaptation of the SoAS.

At last, intercorrelations among F-SoAS factors were also consistent with those reported by Tapal et al. (2017). The weak-to-moderate, negative correlation between SoPA and SoNA suggests that the two factors do not measure the same construct but, rather, measure different aspects of the same construct. Furthermore, our results support a conceptualization of SoA as a complex, multidimensional construct that can be measured by direct methods either on a local, specific dimension, or on a global dimension.

### Gender Differences

The invariance of the F-SoAS across genders datasets supports the idea that it can be used and interpreted similarly across genders. Concerning the effect of gender on F-SoAS scores, our analysis demonstrates the equivalence of the measurement model across genders. This indicates that men and women interpret the scale in the same way and mobilize a comparable response process. We also explored differences between women and men with respect to overall SoA. Our results, which are in line with the literacy, show that women report lower SoA than men. Women also score lower for SoPA (i.e., judgment of having control) and higher for SoNA (i.e., existential helplessness) than men. These results could be due to the smaller number of men than women used in the present study and the subsequent lower power of the study when examining men. However, if our finding is not due to a lack of power, they fit adequately with previous studies on gender effect in agency, even concerning the small effect size (Donald et al., 2017). In all cases, we would call for replications of this effect to evaluate that. If this gender effect turns out to be robust then possible explanations for why and how the gender could influence, or be influenced by, the authorship judgment must be addressed. For now, such explanations are necessarily speculative and will need to be experimentally tested in future studies. One potential explanation comes from Wegner and Wheatley (1999), stating that the experience of conscious will is an expression of our tendency to take the “intentional stance.” This stance consists of viewing “psychological causation not in terms of causal mechanism but rather in terms of agents who have beliefs and desires that cause their acts. Conscious will is part of taking an intentional stance toward oneself” (Wegner and Wheatley, 1999, p. 490). However, studies have argued that this empathizing is greater in women than men (Hall, 1978; Tannen, 1990; Baron-Cohen, 2005; Davis, 2018). It, hence, seems plausible that the greater propensity in women to take the intentional stance toward others may lead to overestimate the agency in other and, by contrast, underestimate their own agency. Another potential explanation comes from the Dual Perspective model, proposing that there are universal perceptions of the self, other persons, and social groups as either agentic content or communal content. The agentic content refers to goal-achievement and task functioning (competence, assertiveness, and decisiveness); the communal content refers to the maintenance of relationships and social functioning (helpfulness, benevolence, and trustworthiness; Cuddy et al., 2008; Paulhus and Trapnell, 2008; Abele and Wojciszke, 2014). However, men are described/prescribed as agentic (power, autonomy, decisive, dominant, and aggressive) and women as communal (empathic, emotional, socializing, and dependent) by gender roles (Smith et al., 2019). These gender stereotypes could help explain why women judge herself as less agentic than man do. Therefore, we would call for our findings to be replicated by other studies using the SoAS, perhaps including independent measures of propensity to take the intentional stance (Jones et al., 2008) or including personality questionnaire for the perception of self and other (for possible measure, see Abele and Wojciszke, 2014). Finally, it would be important to also use the SoAS with different age and culture groups because of the cross-effect of gender, age, and culture (Donald et al., 2017)

### Limitations and Futures Studies

There are some limitations to the current study. First, although the psychometric properties of the F-SoAS were acceptable, the construct validity show low index. This weakness in the validity of the model could be due to the low number of items in the scale (7-item), – i.e., reliability index inflating with the number of items – as well as some item headings to re-precise that may have impacted the response process among respondents. At this step, it could be, thus, interesting to conduct qualitative interviews in order to identify and adjust remaining translation or adaptation issues. Moreover, as recommended, this work would require replication on cross-validation in new French populations.

A second limitation is that we did not directly translate the SoAS from Hebrew but from the English translation given by the original authors (Tapal et al., 2017). Therefore, a validity study of this English translation with a native English-speaking sample would reinforce our results and extend the scope of the SoAS. A third limitation is the absence of comparison between the F-SoAS scores and the others, related, constructs enumerate in the introduction (e.g., Self-Efficacy; Determinism…). Indeed, as the scale was only validated in Hebrew with an Israel population, it would have allowed for a confirmation of distinctions between the SoA measure by the scale and the other constructs. In addition, further psychometric studies with a bigger sample are needed to build standard calibration materials tailored to male and female populations.

Concerning future work, the first avenue of interest would be to continue investigating the effects of socio-demographic criteria (age, academic achievement, employment, etc.,) on SoAS factors. The literature on agency supports the idea that such socio-demographic criteria have an effect (Valås, 2001; Jones et al., 2008; Jejeebhoy et al., 2010; Donald et al., 2017) and replicating these effects would consolidate our scale. In addition, it would be interesting to use the F-SoAS to make a direct distinction between positive and negative aspects of SoA, as a function of socio-demographic criteria.

A second avenue for future research would be to replicate the results of Oren et al. (2016) and Tapal et al. (2017), who reported a close correlation between SoNA and self-reported degree of obsessive–compulsive symptoms, and investigate a possible correlation between SoAS factors and positive symptoms of schizophrenia (hallucinations and delusions). People with obsessive–compulsive disorder seem to have a distorted feeling of agency (Gentsch, et al., 2012; Oren et al., 2016) as do people with schizophrenia (Maeda et al., 2013). Exploring the dissociation between the feeling of agency (a low-level process) and the judgment of agency (a high-level process) in schizophrenia (Fletcher and Frith, 2009; Jeannerod, 2009) based on a new definition of the judgment of agency could help in developing interventions.

Another avenue would be to investigate possible links between religiosity and SoA. Indeed, authors have been investigating how the relation a person has with a god could be used to explain causes of behavior and events for that person. For example, Spilka et al. (1985) have shown the possibility to integrate the religiosity into the attribution theory. A theory explaining how people combine information to form a causal judgment. Religiosity was also studied in perspective with well-being. Carlucci et al. (2015) have shown relationship between religiosity and well-being, which could also be mediated by the locus of control (Fiori et al., 2006). These aspects being conceptually close to the SoA (Feldman, 2017), it would be of particular interest that future studies explore links between religiosity and SoA. By, for example, combining the SoAS with the RFS-12 (Altemeyer and Hunsberger, 2004).

Additionally, any future research on agency that uses this scale would benefit from the addition of indirect measures (intentional binding, sensorial attenuation, etc.,). This would make it possible to investigate any bias associated with self-report measures (for a general discussion about the relationship between implicit measures and verbal reporting, see Gawronski et al., 2007) and address the sometimes-conflicting results of studies that use direct and indirect measures. Although they may seem to be complementary notions, it is relevant to ask whether the feeling of agency (indirect measures) is a precondition for the judgment of agency (direct measures)? Or, on the other hand, is the feeling of agency a subsequent abstraction – a stripped-down version of the original sense (i.e., judgment) of agency? Zahavi (2008) looked at this question into a broader philosophical perspective. More specifically, research needs to go beyond only using judgment, and seek to understand changes in the link with the feeling of agency.

Overall, it would be particularly interesting to use the F-SoAS in learning research. Although the relation between SoA and learning is a promising topic (Chatman and Sparrow, 2011), the notion remains difficult to interpret and studies do not use a global measure of SoA. In this context, a first step is to clarify the links between perceptual effects (indirect measures) and the subjective judgment of being an agent (direct measures), before using responses to the F-SoAS to adapt pedagogical processes to learners’ needs and feelings.

## Conclusion

To conclude, the present study, using factor analysis, provided a validation for the F-SoAS as a short scale of 7-item. The F-SoAS extend a new way to quantify experience of agency (SoAS) for the French population and will help future studies to investigate effects of socio-demographic criteria (age, gender, culture…) on the SoA. Our findings of a gender effect points to the need for further examination of potential effects in the SoA. We have speculated that gender differences in the tendency to take the intentional stance and/or gender stereotypes may extend to the generation of our attributional judgment. In addition, such a methodology should potentially allow a range of future experiments to explore further links between the general, cross-situational, SoA, and learning mechanisms in general population or pathology symptoms (hallucination, Obsessive-Compulsive Symptoms…) in clinical populations. Finally, it should be noted that further cross-validation is required in accordance with recommended standards for test construction and adaptation (American Psychological Association, American Educational Research Association and National Council on Measurements Used in Education, 1954; American Educational Research Association, American Psychological Association, and National Council of Measurement in Education-AERA, APA, and NCME, 2014; International Test Commission, 2014).

## Data Availability Statement

The raw data supporting the conclusions of this article will be made available by the authors, without undue reservation.

## Ethics Statement

Ethical review and approval was not required for the study on human participants in accordance with the local legislation and institutional requirements. The patients/participants provided their written informed consent to participate in this study.

## Author Contributions

J-CH, LB, and AT participated in the design of the study. J-CH conducted the experiment. J-CH, LC, GB, and LB analyzed the results and wrote the manuscript. All authors contributed to the article and approved the submitted version.

### Conflict of Interest

The authors declare that the research was conducted in the absence of any commercial or financial relationships that could be construed as a potential conflict of interest.

## Footnotes

^1^https://www.epsylab.fr
